# Growth hormone associated with treatment efficacy of immune checkpoint inhibitors in gastric cancer patients

**DOI:** 10.3389/fonc.2022.917313

**Published:** 2022-08-09

**Authors:** Yue Zhao, Zhengzheng Ji, Jiasong Li, Shasha Zhang, Chensi Wu, Ruixing Zhang, Zhanjun Guo

**Affiliations:** ^1^ Department of Gastroenterology and Hepatology, The Fourth Hospital of Hebei Medical University, Shijiazhuang, China; ^2^ Department of Immunology and Rheumatology, The Fourth Hospital of Hebei Medical University, Shijiazhuang, China

**Keywords:** gastric cancer, growth hormone, immune checkpoint inhibitors, anti-PD-1 antibody, therapeutic efficacy, prognosis

## Abstract

**Background:**

Immune checkpoint inhibitors (ICIs) combined with chemotherapy have been widely employed to improve the outcome of gastric cancer patients. In the present study, the impact of posttreatment growth hormone (GH) levels on the treatment efficacy of ICIs for advanced gastric cancer (AGC) patients was assessed.

**Methods:**

Seventy-five AGC patients treated with anti-PD-1 antibodies at The Fourth Hospital of Hebei Medical University were involved. We divided AGC patients into two groups as high-GH group and low-GH group based on the GH level. Immunotherapy efficacy was assessed in terms of objective response rate, disease control rate (DCR), progression-free survival (PFS), and overall survival (OS) based on the National Comprehensive Cancer Network Guidelines. The enumeration data were compared by *χ* ^2^ test or Fisher’s exact test. Survival curves were drawn by the Kaplan–Meier method, and comparisons between the curves were made using the log-rank test. Multivariate survival analysis was performed using a Cox proportional hazards model.

**Results:**

The higher GH levels were associated with a lower DCR of ICIs with a DCR of 30.0% in the high-GH group and 53.3% in the low-GH group (*P* = 0.046). The subsequent univariate analysis showed that a high GH level was associated with both shorter PFS (*P* = 0.016) and shorter OS at the borderline statistical level (*P* = 0.052) in AGC patients treated with ICIs. Cox model analysis also proved that the GH level was an independent risk factor for the outcome of AGC patients (PFS: *P* = 0.013, HR, 2.424, 95% CI, 1.202–4.890; OS: *P* = 0.014, HR, 3.301, 95% CI, 1.279–8.519).

**Conclusions:**

The post-treatment GH level might be a predictor for ICIs treatment in AGC patients.

## Introduction

Gastric cancer (GC) is a common lethal malignant tumor worldwide. According to latest data published by GLOBOCAN 2020, there were more than 1 million new cases of GC with 769,000 deaths, ranking the fifth for the incidence and the fourth for the mortality of malignant tumors around the world (1). Risk factors for GC include *Helicobacter pylori* infection, alcohol abuse, smoking, and pickled foods (1). Surgical resection remains the primary treatment means for GC; other methods include chemotherapy, radiotherapy, targeted therapy, and immunotherapy. Traditional chemotherapy drugs exhibit a less ideal effect whereas targeted therapies have limited indication, which gives a poor overall prognosis for advanced gastric cancer (AGC) patients. Immune checkpoint inhibitors (ICIs) enhance antitumor activity by blocking immune-intrinsic downregulating factors such as cytotoxic T-lymphocyte antigen 4 (CTLA-4), programmed cell death 1 (PD-1), and programmed cell death ligand 1 (PD-L1), which made breakthroughs in the treatment of a series of tumors such as gastric cancer, liver cancer, and non-small cell lung cancer (2–4). PD-1 or PD-L1 antibodies could block the PD-1/PD-L1 pathway and positively regulate the activation and function of T lymphocytes, thereby inhibiting tumor immunity, enhancing antitumor immunity, and inhibiting tumor growth (3, 4). Based on the studies of CheckMate-649 and ATTRACTION-4 for significantly improved progression-free survival (PFS), objective response rate (ORR), and disease control rate (DCR), an anti-PD-1 antibody of nivolumab combined with chemotherapy has been recommended as the first-line treatment for AGC (4, 5). Other anti-PD-1 antibodies also displayed beneficial effects in combination with chemotherapy or targeted therapy in clinical trials for AGC treatment (6–8). Despite the breakthroughs of ICIs in the treatment of AGC, only a few biomedical predictors of immunotherapy efficacy such as mismatch repair (MMR)/microsatellite instability (MSI), PD-L1 expression, and gut microbiota have been identified (9–11) previously.

Growth hormone (GH) is a protein hormone secreted by eosinophils in the anterior pituitary gland, which not only binds specifically to the growth hormone receptor (GHR) of the target tissue but also is involved in the regulation of cell proliferation and differentiation by inducing the release of insulin-like growth factor (IGF) as well as mediating signal transduction for Janus kinase 2 (JAK2), mitogen-activated protein kinase (MAPK), and signal transducer and activator of transcription (STAT) (12, 13). As a growth factor with potential capacity to promote tumor cell mitosis and growth, GH could regulate the tumor microenvironment (TME), epithelial–mesenchymal transition (EMT), DNA damage repair, tumor vascular distribution, and chemotherapy resistance, thereby initiating the occurrence and development of tumors (14–16). Recent studies also showed that GH could mediate the expression of PD-1/PD-L1 and regulate CD4^+^ and CD8^+^ T lymphocytes, which might modify the immunotherapy efficacy of tumors by mediating the PD-1/PD-L1 pathway and/or TME (17–19). In the present study, the predictive value of GH for ICI efficacy in AGC patients is innovatively evaluated.

## Methods

### Patients

AGC patients treated with anti-PD-1 antibody monotherapy or combination with chemotherapy or targeted therapy at The Fourth Hospital of Hebei Medical University from January 2019 to April 2022 were involved. Patients who were previously treated with other immunotherapies or had high GH-related underlying diseases were excluded. All clinical data including age, gender, Eastern Cooperative Oncology Group Performance Status (ECOG PS), human epidermal growth factor receptor 2 (HER2) status, MSI status, combined positive score (CPS), Epstein–Barr virus (EBV) status, surgical history, histological type, TNM stage, treatment lines, treatment regimen, disease status, immune-related adverse events (irAE), baseline GH level, and posttreatment GH level were collected for analysis. GH levels of fasting blood samples were measured using the electrochemiluminescence method on a Roche Cobas e602 analyzer with a supporting Elecsys hGH kit and calibrator (Roche, Basel, Switzerland), and all samples were analyzed twice in one assay. The baseline GH level was the GH test value of the first hospitalization before immunotherapy. If the result was abnormal, we repeated the measurement the following day and took the average. The posttreatment GH level was emphasized as the main research factor of this study and was defined as the mean of two hospitalization GH tests after the initial immunotherapy. A high posttreatment GH level means that the GH value exceeds the upper limit of the reference value and occurs twice or more on different days. Low and high GH levels were defined as <2.47 and ≥2.47 ng/ml, respectively, while the watershed is a normal threshold for a healthy person. All procedures were reviewed and approved by the Ethics Committee of the Fourth Hospital of Hebei Medical University (No. 2021136). Informed consent was obtained for all participating patients.

### Treatment and assessment

Patients received standard anti-PD-1 antibodies (monotherapy or combination with targeted/chemotherapy drugs) every 21 days until disease progression, clinical worsening, unacceptable toxicity, and patient refusal. **Table S1** lists treatment lines, treatment duration, number of patients in each therapeutic schedule, and types of immunotherapy drugs, targeted drugs, and chemotherapy drugs. After initiation of treatment, clinical and laboratory tests were performed as clinically indicated in each cycle prior to dosing. Body computed tomography or magnetic resonance imaging scans were performed in every 2–3 cycles. The study endpoints were PFS, overall survival (OS), and the response, which were evaluated using the Response Evaluation Criteria in Solid Tumors version 1.1 criteria.

### Statistical analysis

Statistical analysis was carried out with SPSS 21.0 software (IBM SPSS, NY, USA). PFS was defined as the time interval from the first application of anti-PD-1 therapy to progression, death, or study cutoff. OS was defined as the time interval from commencement of ICI-based systemic therapy to death or study cutoff. The enumeration data were compared by the *χ* ^2^ test or Fisher’s exact test. Survival curves were drawn by the Kaplan–Meier method, and the association between clinical features and survival was analyzed by the log-rank test. Multivariate survival analysis was performed using a Cox proportional hazard model, and *P* < 0.05 was considered statistically significant.

## Results

### Patient characteristics

A total of 75 AGC patients treated with anti-PD-1 antibodies were identified as study subjects, among whom only one patient was treated with ICI monotherapy (toripalimab) and the other 74 were treated with combination therapies (13 patients were treated with triple therapy of immunotherapy, chemotherapy, and targeted therapy, 14 patients received immunotherapy combined with targeted therapy, and 47 patients were treated with immunotherapy plus chemotherapy, **Table S1**). Clinical features are listed in **Table 1**; the mean OS and mean PFS for overall patients were 18.887 months (95% CI: 16.317 to 21.456 months) and 11.740 months (95% CI: 9.053 to 14.428 months), respectively. As for clinical efficacy evaluation, one patient was observed with complete response (CR), 10 with partial response (PR), and 22 with stable disease (SD) (**Table 2**), which resulted in an ORR of 14.7% (95% CI: 6.5% to 22.9%) and a DCR of 44.0% (95% CI: 32.5% to 55.5%).

**Table 1:** Characteristics of AGC patients with different posttreatment GH levels.

	Group	Total no. (%)	High GH group no. (%)	Low GH group no. (%)	*P*
Total N	–	75	30	45	–
Gender	Male	55 (73.3)	22 (73.3)	33 (73.3)	1.000
	Female	20 (26.7)	8 (26.7)	12 (26.7)	
Age	<60	29 (38.7)	15 (50.0)	14 (31.1)	0.100
	≥60	46 (61.3)	15 (50.0)	31 (68.9)	
ECOG PS	0-1	31 (41.3)	11 (36.7)	20 (44.4)	0.503
	2-3	44 (58.7)	19 (63.3)	25 (55.6)	
HER2 status	Negative	66 (88.0)	28 (93.3)	38 (84.4)	0.425
	Positive	9 (12.0)	2 (6.7)	7 (15.6)	
MSI status	MSS/MSI-L	71 (94.7)	29 (96.7)	42 (93.3)	0.916
	MSI-H	4 (5.3)	1 (3.3)	3 (6.7)	
CPS	<5	56 (74.7)	23 (76.7)	33 (73.3)	0.745
	≥5	19 (25.3)	7 (23.3)	12 (26.7)	
EBV status	Negative	67 (89.3)	29 (96.7)	38 (84.4)	0.194
	Positive	8 (10.7)	1 (3.3)	7 (15.6)	
TNM stage	III	24 (32.0)	6 (20.0)	18 (40.0)	0.069
	IV	51 (68.0)	24 (80.0)	27 (60.0)	
Surgical history	No	51 (68.0)	16 (53.3)	35 (77.8)	0.026
	Yes	24 (32.0)	14 (46.7)	10 (22.2)	
IrAE	No	54 (72.0)	24 (80.0)	30 (66.7)	0.208
	Yes	21 (28.0)	6 (20.0)	15 (33.3)	
Treatment lines	1-2	57 (76.0)	20 (66.7)	37 (82.2)	0.122
	≥3	18 (24.0)	10 (33.3)	8 (17.8)	
Treatment regimen	Monotherapy/duplex-therapy	62 (82.7)	23 (76.7)	39 (86.7)	0.262
	Triple-therapy	13 (17.3)	7 (23.3)	6 (13.3)	
Serum albumin (g/L)	<30	49 (65.3)	22 (73.3)	27 (60.0)	0.235
	≥30	26 (34.7)	8 (26.7)	18 (40.0)	

AGC, advanced gastric cancer; GH, growth hormone; ECOG PS, Eastern Cooperative Oncology Group Performance Status; HER2, human epidermal growth factor receptor 2; MSI, microsatellite instability; CPS, Combined Positive Score; EBV, Epstein–Barr virus; irAE, immune-related adverse events.

**Table 2:** Effect of posttreatment GH levels on ICI efficacy.

Response	Total	High GH group	Low GH group	*P*
PD	42	21	21	–
SD	22	7	15	–
PR	10	2	8	–
CR	1	0	1	–
ORR	14.7% (95% CI: 6.5%-22.9%)	6.7% (95% CI: -2.8%-16.1%)	20.0% (95% CI: 7.8%-32.2%)	0.206
DCR	44.0% (95% CI: 32.5%-55.5%)	30.0% (95% CI: 12.6%-47.4%)	53.3% (95% CI: 38.2%-68.5%)	0.046

PD, progressive disease; SD, stable disease; PR, partial response; CR, complete response; ORR, objective response rate; DCR, disease control rate; GH, growth hormone.

### Growth hormone associated with DCR in AGC patients

The age and gender of AGC patients with different baseline and posttreatment GH levels were compared, respectively; there was no significant difference for distribution frequency referring to age and gender between high- and low-GH groups (*P* > 0.05, **Table 1** and **Table S2**), which indicated that the groups were balanced and comparable. The clinical characteristics including ECOG PS, HER2 status, MSI status, CPS, EBV status, TNM stage, irAE, treatment lines, treatment regimen, and serum albumin were not associated with posttreatment GH expression status except for surgical history (*P* = 0.026, **Table 1**). In addition, all the above clinical characteristics were not correlated with baseline GH levels (*P* > 0.05, **Table S2**). The ORRs of the high and low posttreatment GH groups were 6.7% and 20.0% (*P* = 0.206), but the DCR distribution was significantly different with 30.0% for the high-GH group and 53.3% for the low-GH group (*P* = 0.046, **Table 2**). These results demonstrated that GH might affect the treatment efficacy of anti-PD-1 antibodies in AGC patients.

### Growth hormone associated with PFS and OS in AGC patients

**Figure 1:**
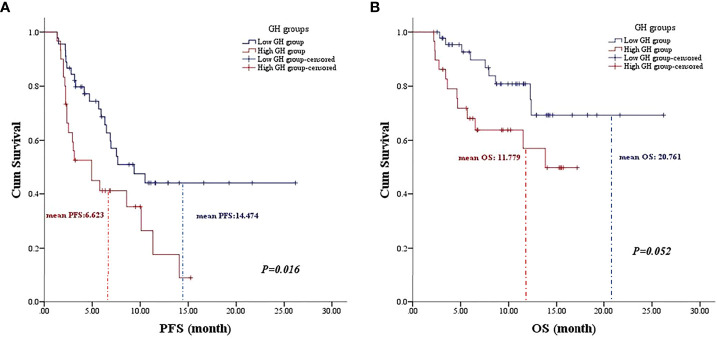
The association of posttreatment growth hormone levels on the prognosis of gastric cancer patients. **(A)** The Kaplan–Meier curve of progression-free survival. **(B)** The Kaplan–Meier curve of overall survival.

Univariate analysis was performed with the clinical features that might affect the PFS of gastric cancer. As shown in **Table 3**, the mean PFS of 14.474 months (95% CI: 11.030 to 17.917 months) for the low post-treatment GH group was significantly extended compared with the mean PFS of 6.623 months (95% CI: 4.718 to 8.528 months) for the high posttreatment group (*P* = 0.016, **Figure 1A**). Meanwhile, the low baseline GH group with a mean PFS of 12.471 months (95% CI: 9.620 to 15.321 months) displayed a prolonged PFS than that of the high group with a mean PFS of 3.467 months (95% CI: 2.124 to 4.810 months) at significant statistical levels (*P* = 0.004). In addition, the treatment lines (P = 0.009, mean PFS 12.596 vs. 5.773 months), ECOG PS (*P* = 0.000, mean PFS 17.464 vs. 6.271 months), and TNM stage (*P* = 0.007, mean PFS=13.602 vs. 9.010 months) were associated with PFS in these AGC patients, whereas gender, age, HER2 status, MSI status, CPS, EBV status, surgical history, irAE, treatment regimen, and serum albumin had no significant effect on PFS (*P* > 0.05). In multivariate analysis, a high posttreatment GH level was independently associated with a poor PFS (*P* = 0.013, HR: 2.424, 95% CI: 1.202–4.890, **Table 4**). The ECOG PS (*P* = 0.001, HR: 4.769, 95% CI: 1.973–11.529), TNM stage (*P* = 0.018, HR: 2.804, 95% CI: 1.197–6.571), surgery history (*P* = 0.020, HR: 0.356, 95% CI: 0.149–0.847), and treatment lines (*P* = 0.001, HR: 3.932, 95% CI: 1.793–8.621) were proved to be independently associated with PFS (**Table 4**).

**Table 3:** Univariate analyses of progression-free survival and overall survival.

Covariate	Group	PFS			OS		
		HR	95%CI	*P*	HR	95%CI	*P*
Gender	Male	Reference	0.515-1.972	0.981	Reference	0.318-2.138	0.692
	Female	1.008			0.825		
Age	<60	Reference	0.781-2.896	0.223	Reference	0.581-3.873	0.401
	≥60	1.504			1.501		
ECOG PS	0-1	Reference	1.789-7.574	0.000	Reference	1.039-9.227	0.043
	2-3	3.681			3.096		
HER2 status	Negative	Reference	0.505-2.858	0.679	Reference	0.337-3.887	0.829
	Positive	1.201			1.144		
MSI status	MSS/MSI-L	Reference	0.210-3.611	0.849	Reference	0.131-7.333	0.985
	MSI-H	0.871			0.981		
CPS score	<5	Reference	0.320-1.504	0.354	Reference	0.247-2.223	0.592
	≥5	0.693			0.741		
EBV status	Negative	Reference	0.211-1.675	0.325	Reference	0.144-2.672	0.522
	Positive	0.595			0.621		
TNM stage	III	Reference	1.353-6.411	0.007	Reference	0.942-10.865	0.062
	IV	2.945			3.199		
Surgical history	No	Reference	0.299-1.196	0.146	Reference	0.043-0.809	0.025
	Yes	0.598			0.187		
IrAE	No	Reference	0.384-1.477	0.409	Reference	0.137-1.225	0.110
	Yes	0.753			0.409		
Baseline GH level	Low level	Reference	1.502-9.025	0.004	Reference	0.998-9.053	0.050
	High level	3.682			3.007		
Posttreatment GH levels	Low level	Reference	1.151-3.892	0.016	Reference	0.993-5.615	0.052
	High level	2.116			2.362		
Treatment lines	1-2	Reference	1.235-4.527	0.009	Reference	1.265-7.177	0.013
	≥3	2.364			3.013		
Treatment regimen	Monotherapy/duplex-therapy	Reference	0.589-2.773	0.535	Reference	0.631-4.741	0.287
	Triple-therapy	1.278			1.729		
Serum albumin (g/L)	<30	Reference	0.477-1.690	0.738	Reference	0.747-4.227	0.193
	≥30	0.898			1.777		

PFS, progression-free survival; OS, overall survival; ECOG PS, Eastern Cooperative Oncology Group Performance Status; HER2, human epidermal growth factor receptor 2; MSI, microsatellite instability; CPS, combined positive score; EBV, Epstein–Barr virus; irAE, immune-related adverse events; GH, growth hormone.

**Table 4:** Multivariate analyses of progression-free survival and overall survival with the Cox proportional hazards model.

	PFS			OS		
	HR	95%CI	*P*	HR	95%CI	*P*
Age	1.150	0.543-2.436	0.716	1.132	0.382-3.355	0.822
ECOG PS	4.769	1.973-11.529	0.001	1.403	0.422-4.662	0.580
TNM stage	2.804	1.197-6.571	0.018	4.050	1.120-14.648	0.033
Surgical history	0.356	0.149-0.847	0.020	0.053	0.009-0.300	0.001
IrAE	1.213	0.573-2.566	0.614	0.419	0.120-1.461	0.172
Posttreatment GH levels	2.424	1.202-4.890	0.013	3.301	1.279-8.519	0.014
Treatment lines	3.932	1.793-8.621	0.001	7.302	2.621-20.344	0.000

PFS, progression-free survival; OS, overall survival; ECOG PS, Eastern Cooperative Oncology Group performance status; irAE, immune-related adverse events; GH, growth hormone.

As for OS, the low posttreatment GH group with a mean OS of 20.761 months (95% CI: 17.663 to 23.859 months) displayed prolonged survival time than that of the high group with a mean OS of 11.779 months (95% CI: 9.409 to 14.149 months) at the borderline statistical level (*P* = 0.052, **Table 3**, **Figure 1B**). Similarly, this survival advantage for OS in the low baseline GH group was also obtained at a critically statistical level (*P* = 0.050, mean OS 19.578 vs. 8.511 months). Besides, ECOG PS (*P* = 0.043, mean OS 22.274 vs. 12.717 months), surgical history (*P* = 0.025, mean OS 16.754 vs. 20.116 months), and treatment lines (*P* = 0.013, mean OS 20.761 vs. 10.059 months) were also significantly correlated with the OS of AGC patients (**Table 3**), whereas the TNM stage group was associated with OS at the critically statistical level (*P* = 0.062) by univariate analysis. The multivariate analysis indicated that posttreatment GH levels (*P* = 0.014, HR: 3.301, 95% CI: 1.279–8.519), TNM stage (*P* = 0.033, HR: 4.050, 95% CI: 1.120–14.648), surgical history (*P* = 0.001, HR: 0.053, 95% CI: 0.009–0.300), and treatment lines (*P* = 0.000, HR: 7.302, 95% CI: 2.621–20.344) were independent factors for the OS of AGC patients (**Table 4**). The concluded results suggest that posttreatment GH level is an independent factor that affects prognosis in AGC patients, and the risk of death in the high GH level group is 3.301 times higher than that in the low GH level group.

## Discussion

The treatment of advanced gastric cancer has undergone a transition from chemotherapy to targeted therapy, and further to immunotherapy. Clinical research related to immunotherapy has also moved closer from the third-line to first-line treatment. Although GH could increase proliferation for some cancers (20–22), GH testing has not been performed routinely and included in the tumor examination procedure in the past decade. However, with a wide application of ICIs and gradual in-depth understanding of irAE, endocrine-related indexes, including GH levels, are highly valued in cancer patients receiving immunotherapy. According to National Comprehensive Cancer Network (NCCN) Guidelines for Management of Immunotherapy-Related Toxicities Version 1.2022, pituitary and adrenal function monitoring could be considered for cancer patients undergoing immunotherapy (23). With the focus on hypophysitis of irAEs, we routinely tested the pituitary function test (including GH level) of some AGC patients receiving immunotherapy with the consent of patients. Combining these elevated baseline and posttreatment GH level data, we found that the posttreatment GH level was related to DCR, and the baseline and posttreatment GH levels were largely related to prognosis in AGC patients with ICI treatment. The rise in GH level at baseline and posttreatment might represent different meanings. According to the results of survival analysis, we speculate that immunotherapy efficacy and prognosis in AGC patients with a high baseline GH level might be worse. Due to the small number of cases with elevated baseline (7 cases), larger sample size and more in-depth studies are needed to analyze the relationship between GH and gastric cancer.

At present, the mechanism of how GH levels mediate the efficacy of ICIs in tumor patients is still unclear. GH/GHR could mediate the TME and signal transduction including JAK/STAT, MAPK/phosphatidyl inositol 3 kinase (PI3K)/Akt, matrix metallopeptidase 2, and VEGF/VEHGR in gastric cancer, breast cancer, and melanoma (16, 20–22). A growing number of studies have found that these tumor classical signaling pathways were related to the efficacy of ICIs (24–27). The JAK/STAT pathway could transmit the cytokine-mediated signals, increase the expression of PD-1/PD-L1, and reduce the activity of immune cells so as to decrease the body’s antitumor immunity in head and neck cancer (24). This pathway also impaired cytotoxic T lymphocyte to reduce the efficacy of ICIs by initiating chronic inflammation in pancreatic cancer cells (25). The MAPK and PI3K signaling pathways were essential for PD-L1 gene expression, thereby the MAPK inhibitor was used to increase the efficacy of anti-PD-1/PD-L1 inhibitors in melanoma (26, 27). GH might weaken the ICI efficacy by mediating these signaling pathways in AGC patients.

In addition to its effect on the immune microenvironment, GH could also directly promote the uncontrolled proliferation of transformed cells through potential autocrine and/or paracrine pathways (16, 28–30). The GH–GHR–IGF axis increases tumor angiogenesis in GC. GH acts directly on tumor vascular endothelial cells, while GHR is significantly expressed in the vascular endothelium, especially in neovascularization neocapillaries (16, 28). Moreover, GH-IGF could increase VEGF expression and promote tumor angiogenesis *via* PI3K/Akt and MEK/ERK signaling pathways (29, 30). The tumor angiogenesis by GH might make AGC patients resistant to ICIs combined with chemotherapy or targeted therapy (31). Excessive GH creates a pro-tumor environment to the accumulation of oncogenic mutations and chromosomal instability by inhibiting tumor-suppressor proteins and the DNA repair system (32, 33). GH could induce or exacerbate EMT in TME through NF-kappaB signaling to facilitate breast cancer metastasis as well (14).

In recent years, immunotherapy-induced anterior hypophysitis with inhibited adrenocorticotropic hormone and thyroid-stimulating hormone secretion has always been the focus of clinicians’ attention, while the change in GH level after immunotherapy is often overlooked. In this study, GH levels after immunotherapy in 40% of the enrolled AGC patients (30/75) were elevated, which might be related to the immune activation of ICIs, while the underlying mechanisms remain unknown. Kanie et al. (34) retrospectively analyzed 20 patients with PD-1/PD-L1 inhibitor-related hypophysitis and pointed out that anti-pituitary antibody, anti-corticotroph antibody, and anti-somatotroph antibody were exhibited in a portion of these patients. The role of these autoantibodies still needs further studied. It is important to note that pituitary adenoma and extrapituitary diseases (hypothalamic hamartoma, bronchial carcinoid, etc.) could cause the pathological elevation of GH, while stress, exercise, and malnutrition might have a bearing on the physiological increase in serum GH level. Therefore, a single random GH level might be a confounding factor in studies with a small sample size. Researchers might need to avoid errors by detecting the GH level on different days. Conditional medical institutions could comprehensively evaluate the levels of GH and IGF-1, so as to better determine GH hypersecretion. In addition, gastric cancer is one of the tumors with the highest nutritional risk. Among the studies of GH level and gastric cancer, researchers should pay close attention to the nutritional status of such patients by detecting serum albumin and the Nutritional Risk Screening 2002 (NRS2002) or Patient-generated Subjective Global Assessment (PG-SGA) score, so as to avoid malnutrition-related GH elevation as a confounding factor (35).

This study also has some limitations that could be improved. Firstly, it is a single-center retrospective study, and data from multiple centers would be evaluated in further studies. Secondly, limited by our current condition, the sample size of this study was small, which might lead to large confidence intervals and affect the imprecision of the results. The larger sample size is valuable for stratified analysis to identify the effect of GH on ICI efficiency in different surgical history subgroups. Thirdly, there are some potential limitations in regarding ORR as an endpoint in the study of ICI efficacy, because it might miss AGC patients who continuously obtain stable results from immunotherapy and bring long-term survival, and it is not a comprehensive measure of clinical benefit. Therefore, this study not only applied ORR and DCR but also integrated OS and PFS results to evaluate the effect of GH level on treatment efficacy of ICIs in AGC patients, which makes the overall conclusion more comprehensive and reliable. Fourthly, the side effect of combined medicine was not completely excluded in this study, although the combination with medicines such as trastuzumab, lenvatinib, apatinib, and chemotherapeutic agents often displayed different side effects compared with ICIs for GC treatment and rarely showed endocrine toxicity. Endocrinopathies including hypophysitis, thyroid dysfunction, and adrenal insufficiency are among the most common irAEs associated with ICI treatment. However, to the best of our knowledge, this is the first study to reveal the relationship between GH and the ICI efficacy in the treatment of gastric cancer. The influence of GH on ICI treatment of tumor seems to be gastric cancer specific; we evaluated its effect on hepatocellular carcinoma and esophageal cancer patients with ICI treatment, but no association could be found (**Table S3**).

In conclusion, GH might be used as a predictive marker for ICI therapy in AGC patients.

## Data availability statement

The original contributions presented in the study are included in the article/**Supplementary Material**. Further inquiries can be directed to the corresponding author.

## Ethics statement

The studies involving human participants were reviewed and approved by the Ethics Committee of the Fourth Hospital of Hebei Medical University (No. 2021136). The patients/participants provided their written informed consent to participate in this study.

## Author contributions

YZ and ZG contributed to the conceptualization and study design; ZJ, CW, and JL collected the patient data; YZ, ZJ, and JL performed the statistical analysis; YZ and SZ wrote the original draft of the manuscript; RZ and ZG administrated and supervised the project; all authors contributed to manuscript revision and read and approved the submitted version.

## Funding

This study was funded by a grant from the S&T Program of Hebei Province (number 20377726D).

## Conflict of interest

The authors declare that the research was conducted in the absence of any commercial or financial relationships that could be construed as a potential conflict of interest.

## Publisher’s note

All claims expressed in this article are solely those of the authors and do not necessarily represent those of their affiliated organizations, or those of the publisher, the editors and the reviewers. Any product that may be evaluated in this article, or claim that may be made by its manufacturer, is not guaranteed or endorsed by the publisher.
