# Supplementary material for: Growth hormone associated with treatment efficacy of immune checkpoint inhibitors in gastric cancer patients
Source: Front Oncol. 2022 Aug 9;12:917313. doi: 10.3389/fonc.2022.917313 (PMC9395680; doi:10.3389/fonc.2022.917313)
Supplement: Supplementary file 1 [file Table_1.docx]

Supplementary Material

Table S1. Treatment information of patients with advanced gastric cancer.

| Treatment lines | Therapeutic schedule | | | No. of patients | Treatment duration (months) |
| --- | --- | --- | --- | --- | --- |
|  | ICIs | Targeted drug | Chemotherapeutic regimen |  |  |
| 1 | Camrelizumab | Trastuzumab | Oxaliplatin/ Lobaplatin+S-1 | 1 | 6.27 |
| 1 | Camrelizumab | - | Oxaliplatin/ Lobaplatin+Capecitabine | 1 | 7.83 |
| 1 | Camrelizumab | - | Oxaliplatin/ Lobaplatin+S-1 | 5 | 3.85±0.83 |
| 1 | Camrelizumab | - | S-1 | 1 | 4.20 |
| 1 | Pembrolizumab | Trastuzumab | Paclitaxel+Oxaliplatin+S-1 | 1 | 6.10 |
| 1 | Pembrolizumab | Lenvatinib | - | 1 | 13.77 |
| 1 | Pembrolizumab | - | Lobaplatin+S-1 | 1 | 6.20 |
| 1 | Sintilimab | Apatinib | Paclitaxel+S-1 | 1 | 7.87 |
| 1 | Sintilimab | Trastuzumab | Oxaliplatin/ Lobaplatin+S-1 | 1 | 9.07 |
| 1 | Sintilimab | - | Oxaliplatin/ Lobaplatin+S-1 | 10 | 6.03±3.31 |
| 1 | Sintilimab | - | Paclitaxel+Lobaplatin | 1 | 5.77 |
| 1 | Sintilimab | - | S-1 | 1 | 5.20 |
| 1 | Tislelizumab | Trastuzumab | S-1 | 1 | 6.80 |
| 1 | Tislelizumab | Apatinib | - | 1 | 4.67 |
| 1 | Tislelizumab | - | Oxaliplatin/ Lobaplatin+Capecitabine | 4 | 6.87±2.50 |
| 1 | Tislelizumab | - | Oxaliplatin/ Lobaplatin+S-1 | 5 | 5.89±2.23 |
| 1 | Tislelizumab | - | Paclitaxel+S-1 | 1 | 4.77 |
| 1 | Tislelizumab | - | S-1 | 1 | 6.33 |
| 1 | Toripalimab | - | Capecitabine | 1 | 22.67 |
| 2 | Camrelizumab | Apatinib | Paclitaxel | 2 | 2.80±0.18 |
| 2 | Camrelizumab | Apatinib | - | 3 | 7.77±7.39 |
| 2 | Camrelizumab | Lenvatinib | - | 1 | 3.87 |
| 2 | Camrelizumab | - | Paclitaxel+S-1 | 1 | 9.10 |
| 2 | Pembrolizumab | Trastuzumab | Paclitaxel | 1 | 3.90 |
| 2 | Pembrolizumab | - | Paclitaxel+Fluorouracil | 1 | 2.33 |
| 2 | Sintilimab | Apatinib | - | 1 | 8.33 |
| 2 | Sintilimab | - | Oxaliplatin/ Lobaplatin+S-1 | 2 | 4.24±0.52 |
| 2 | Sintilimab | - | Oxaliplatin/ Lobaplatin+Capecitabine | 1 | 9.67 |
| 2 | Sintilimab | - | Paclitaxel+S-1 | 3 | 3.94±0.91 |
| 2 | Sintilimab |  | Paclitaxel+Fluorouracil | 1 | 3.57 |
| 2 | Tislelizumab | Lenvatinib | - | 1 | 11.67 |
| 2 | Tislelizumab | - | Paclitaxel+S-1 | 1 | 3.60 |
| 2 | Tislelizumab | - | Paclitaxel | 1 | 3.40 |
| 2 | Tislelizumab | - | Fluorouracil | 1 | 2.97 |
| 2 | Toripalimab | - | S-1 | 1 | 2.87 |
| 3 | Camrelizumab | Apatinib | Paclitaxel | 3 | 4.33±1.87 |
| 3 | Camrelizumab | Apatinib | - | 3 | 2.93±0.33 |
| 3 | Camrelizumab | Lenvatinib | - | 1 | 2.67 |
| 3 | Sintilimab | Apatinib | Irinotecan | 1 | 2.87 |
| 3 | Sintilimab | - | Paclitaxel+S-1 | 1 | 4.67 |
| 3 | Tislelizumab | Apatinib | Lobaplatin+S-1 | 1 | 5.33 |
| 3 | Tislelizumab | - | Capecitabine | 1 | 3.90 |
| 3 | Toripalimab | Apatinib | - | 1 | 6.67 |
| 4 | Toripalimab | Apatinib | - | 1 | 4.80 |
| 4 | Toripalimab | - | - | 1 | 3.20 |

ICIs, immune checkpoint inhibitors

Table S2. Characteristics of AGC patients with different baseline growth hormone levels.

|  | Group | Total no. (%) | High GH group no. (%) | Low GH group no. (%) | *P* |
| --- | --- | --- | --- | --- | --- |
| Total N | - | 75 | 7 | 68 | - |
| Gender | Male | 55 (73.3) | 5 (71.4) | 50 (73.5) | 1.000 |
|  | Female | 20 (26.7) | 2 (28.6) | 18 (26.5) |  |
| Age | <60 | 29 (38.7) | 3 (42.9) | 26 (38.2) | 1.000 |
|  | ≥60 | 46 (61.3) | 4 (57.1) | 42 (61.8) |  |
| ECOG PS | 0-1 | 31 (41.3) | 3 (42.9) | 28 (41.2) | 1.000 |
|  | 2-3 | 44 (58.7) | 4 (57.1) | 40 (58.8) |  |
| HER2 status | Negative | 66 (88.0) | 6 (85.7) | 60 (88.2) | 1.000 |
|  | Positive | 9 (12.0) | 1 (14.3) | 8 (11.8) |  |
| MSI status | MSS/MSI-L | 71 (94.7) | 7 (100.0) | 64 (94.1) | 1.000 |
|  | MSI-H | 4 (5.3) | 0 (0.0) | 4 (5.9) |  |
| CPS | <5 | 56 (74.7) | 7 (100.0) | 49 (72.1) | 0.181 |
|  | ≥5 | 19 (25.3) | 0 (0.0) | 19 (27.9) |  |
| EBV | Negative | 67 (89.3) | 6 (85.7) | 61 (89.7) | 1.000 |
|  | Positive | 8 (10.7) | 1 (14.3) | 7 (10.3) |  |
| TNM stage | Ⅲ | 24 (32.0) | 1 (14.3) | 23 (33.8) | 0.529 |
|  | Ⅳ | 51 (68.0) | 6 (85.7) | 45 (66.2) |  |
| Surgical history | No | 51 (68.0) | 6 (85.7) | 45 (66.2) | 0.529 |
|  | Yes | 24 (32.0) | 1 (14.3) | 23 (33.8) |  |
| IrAE | No | 54 (72.0) | 5 (71.4) | 49 (72.1) | 1.000 |
|  | Yes | 21 (28.0) | 2 (28.6) | 19 (27.9) |  |
| Treatment lines | 1-2 | 57 (76.0) | 5 (71.4) | 52 (76.5) | 1.000 |
|  | ≥3 | 18 (24.0) | 2 (28.6) | 16 (23.5) |  |
| Treatment regimen | Monotherapy/ duplex-therapy | 62 (82.7) | 4 (57.1) | 58 (85.3) | 0.177 |
|  | Triple-therapy | 13 (17.3) | 3 (42.9) | 10 (14.7) |  |
| Serum albumin (g/L) | <30 | 49 (65.3) | 4 (57.1) | 45 (66.2) | 0.951 |
|  | ≥30 | 26 (34.7) | 3 (42.9) | 23 (33.8) |  |

AGC, advanced gastric cancer; GH, growth hormone; ECOG PS, Eastern Cooperative Oncology Group Performance Status; HER2, human epidermal growth factor receptor 2; MSI, microsatellite instability; CPS, Combined Positive Score; EBV, Epstein-Barr virus; irAE, immune related adverse events.

Table S3. Survival analyses of progression-free survival and overall survival in hepatocellular carcinoma and esophageal carcinoma.

| Type of cancer | Patients with low GH | Patients with high GH | PFS | | | OS | | |
| --- | --- | --- | --- | --- | --- | --- | --- | --- |
|  |  |  | HR | 95%CI | *P* | HR | 95%CI | *P* |
| Hepatocellular carcinoma | 13 | 7 | 0.871 | 0.212-3.584 | 0.848 | 0.624 | 0.209-1.857 | 0.396 |
| Esophageal carcinoma | 19 | 3 | 1.668 | 0.352-7.904 | 0.519 | 1.667 | 0.346-8.039 | 0.524 |

GH, growth hormone; PFS, progression-free survival; OS, overall survival.
